# Balancing Microthrombosis and Inflammation via Injectable Protein Hydrogel for Inflammatory Bowel Disease

**DOI:** 10.1002/advs.202200281

**Published:** 2022-05-07

**Authors:** Liwen Hong, Gaoxian Chen, Zhengwei Cai, Hua Liu, Chen Zhang, Fei Wang, Zeyu Xiao, Jie Zhong, Lei Wang, Zhengting Wang, Wenguo Cui

**Affiliations:** ^1^ Department of Gastroenterology Ruijin Hospital Shanghai Jiao Tong University School of Medicine 197 Ruijin 2nd Road Shanghai 200025 P. R. China; ^2^ Department of Orthopaedics Shanghai Key Laboratory for Prevention and Treatment of Bone and Joint Diseases Shanghai Institute of Traumatology and Orthopaedics Ruijin Hospital Shanghai Jiao Tong University School of Medicine 197 Ruijin 2nd Road Shanghai 200025 P. R. China; ^3^ Department of Pharmacology and Chemical Biology Institute of Molecular Medicine School of Medicine Shanghai Jiao Tong University Shanghai 200025 P. R. China; ^4^ Department of Geriatrics Ruijin Hospital Shanghai Jiao Tong University School of Medicine 197 Ruijin 2nd Road Shanghai 200025 P. R. China

**Keywords:** inflammatory bowel disease, microthrombosis, protein hydrogel

## Abstract

Emerging evidence indicates that a vicious cycle between inflammation and microthrombosis catalyzes the pathogenesis of inflammatory bowel disease (IBD). Over‐stimulated inflammation triggers a coagulation cascade and leads to microthrombosis, which further complicates the injury through tissue hypoxia and ischemia. Herein, an injectable protein hydrogel with anti‐thrombosis and anti‐inflammation competency is developed to impede this cycle, cross‐linked by silver ion mediated metal‐ligand coordination and electronic interaction with sulfhydryl functionalized bovine serum albumin and heparin, respectively. The ex vivo experiments show that the hydrogel, HEP‐Ag‐BSA, exhibits excellent self‐healing ability, injectability, biocompatibility, and sustained drug release. HEP‐Ag‐BSA also demonstrates anti‐coagulation and anti‐inflammation abilities via coagulation analysis and lipopolysaccharide stimulation assay. The in vivo imaging confirms the longer retention time of HEP‐Ag‐BSA at inflammatory sites than in normal mucosa owing to electrostatic interactions. The in vivo study applying a mouse model with colitis also reveals that HEP‐Ag‐BSA can robustly inhibit inflammatory microthrombosis with reduced bleeding risk. This versatile protein hydrogel platform can definitively hinder the “inflammation and microthrombosis” cycle, providing a novel integrated approach against IBD.

## Graphical abstract

A microthrombosis and inflammation balancing protein hydrogel, HEP‐Ag‐BSA, is crosslinked by silver ion mediated metal‐ligand coordination and electronic interaction with sulfhydryl functionalized bovine serum albumin and heparin. This injectable hydrogel has prolonged retention at inflamed site, reduces the release of proinflammatory cytokines, and robustly inhibits microthrombosis, which exerts a definitive therapeutic effect on the pathogenesis of inflammatory bowel disease with guaranteed safety.

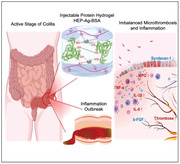

## 1 Introduction

Inflammatory bowel disease (IBD) is an idiopathic chronic inflammatory disorder of the gastrointestinal tract.^[^ ^1^ ^]^ Vicious cycles of inflammation and microthrombosis frequently occur in the pathogenesis of IBD due to the disturbances of immune homeostasis and coagulation.^[^ ^2^, ^3^ ^]^ Stimulated by persistent inflammation at the site of tissue damage in the gut, immune cells continuously release pro‐inflammatory tissue necrosis factor‐*α* (TNF‐*α*), interleukin‐1*β* (IL‐1*β*), interleukin‐6 (IL‐6), nuclear factor‐kappa B (NF‐*κ*B), and so on, thus promoting the expression of tissue factor and activation of plaquettes, which in turn leads to microthrombosis.^[^ ^4^, ^5^ ^]^ The in situ thromboembolism causes blockages in micro‐circulation, resulting in ischemia, hypoxia, and tissue necrosis. This retrospectively boosts the release of pro‐inflammatory factors that exacerbate inflammatory tissue damage.^[^ ^6^ ^]^ The pathological examinations have confirmed the coexistence of vasculitis, mucosal capillary thrombi, and ulceration at primary lesion sites, substantiating the mutual promotion of inflammation and microthrombosis in IBD.^[^ ^7^, ^8^ ^]^ The increasing evidence has indicated that the integration of anti‐inflammation and anti‐thrombosis measures at an early stage is a central component of the therapeutic process in IBD. Nonetheless, the precautions against microthrombosis are not routinely emphasized, which allows for further development of the cycles, and partially explains the recurring outbreaks and protracted courses of IBD.

Conventional treatment for IBD includes steroidal and nonsteroidal anti‐inflammatory drugs; however, they are accompanied by significant dose‐dependent adverse effects such as corticosteroid‐related cognitive alterations and sulfasalazine‐related headache.^[^ ^9^, ^10^ ^]^ Though biologicals have superior treatment outcomes, they may increase the incidence of infections and tumors.^[^ ^11^, ^12^ ^]^ Furthermore, the above‐mentioned agents can hardly modulate microthrombosis. Meanwhile, certain antithrombotics were found to have the ability to act as a potential immunomodulator, but each one has its drawbacks, like aspirin‐induced enteropathy, low absorption of P2Y12 inhibitors in the lumen of the bowel, and lack of clinical evidence for some serine proteases.^[^ ^13^, ^14^, ^15^ ^]^ On the other hand, increasing evidence suggests that heparin, the classical anti‐coagulant, can regulate inflammation in multiple ways, such as inhibition of NF‐*κ*B, suppression of selectin‐induced neutrophil adhesion, and neutralization of inflammatory mediators.^[^ ^16^, ^17^ ^]^ Small scaled clinical trials reported a moderate effect of conventional dosing of heparin and its derivatives for treating colitis.^[^ ^18^ ^]^ However, increased bleeding risk and inferior therapeutic efficacy restrict its application.^[^ ^19^ ^]^ Administration of heparin via the gastrointestinal tract may lower the bleeding risk compared to standard systematic dosing, whereas the high solubility and the low resistance to the physical environment limit the drug concentration at the inflamed site.^[^ ^20^ ^]^ Therefore, optimizing the drug delivery system is essential for the effective use of heparin as a “cycle blocker” in IBD.

Various drug delivery systems have been designed for IBD, including nanoparticles for colon‐targeted or inflammation‐targeted drug delivery, and hydrogels for regeneration of damaged mucosa including a mucosal adhesive hydrogel previously developed by our group.^[^ ^21^, ^22^, ^23^, ^24^, ^25^ ^]^ These inventions help to increase drug bioavailability, reduce side effects, and improve medication adherence. Nonetheless, there are some obvious drawbacks, such as 1) limited drug retention time is not conducive to local lesion healing and requires repeated dosage; 2) in some non‐targeting drug delivery systems, it is difficult to maintain a stable therapeutic environment at the lesion site; 3) the above‐mentioned studies failed to note the impact of anti‐thrombosis on the subsequent development of inflammation. Fortunately, the vast developments in drug delivery systems and engineered regeneration have brought inspiring results.^[^ ^26^, ^27^ ^]^ We exploited the advantages of the native proteins such as bovine serum albumin (BSA) to develop a novel system with good biocompatibility, capable of composing a 3‐dimension (3D) scaffold structure mimicking extracellular matrix (ECM) for tissue regenesis.^[^ ^28^, ^29^ ^]^ Furthermore, since the inflammatory lesions are positively charged due to various cationic exudates, the negatively charged protein hydrogel can prolong lesion site retention via electrostatic attraction, thus directly affecting inflammation modulation.^[^ ^30^, ^31^, ^32^, ^33^ ^]^ Hence, developing a protein hydrogel platform may shed new light on the management of IBD.

**Scheme 1:**
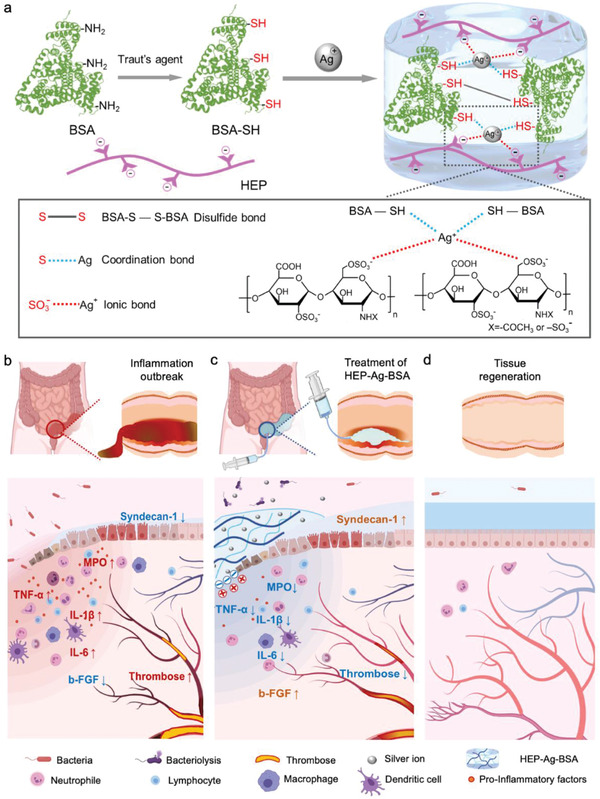
Schematic illustration of the preparation of HEP‐Ag‐BSA and its mode of action in balancing inflammation and microthrombosis in inflammatory bowel disease (IBD). a) Coordinative bond S—Ag, ionic bond SO_3_—Ag^+^, and disulfide bond were formed during the gelation of HEP‐Ag‐BSA. b) The outbreak of IBD featuring mucosal injury and microthrombosis. c) The local administration of HEP‐Ag‐BSA and its mechanism in treating IBD. d) The therapeutic outcomes of HEP‐Ag‐BSA.

To restore the balance of inflammation and microthrombosis in IBD, a heparin‐functionalized protein hydrogel, HEP‐Ag‐BSA, was innovatively constructed based on the dynamic electrostatic attraction between silver ions (Ag^+^) and heparin, with the properties of inhibition of inflammation and microthrombosis, and coordination between Ag^+^ and sulfhydryl functionalized BSA (BSA‐SH) (**Scheme** **1**). According to ex vivo experiments, the novel hydrogel displayed a porous structure, excellent self‐healing property, biocompatibility, and sustained drug release behavior. Benefiting from the therapeutic effects of heparin, HEP‐Ag‐BSA was found to hold the anti‐thrombosis and anti‐inflammation functions, which were further validated by in vitro coagulation and lipopolysaccharide (LPS) stimulation assay. Furthermore, the adhesion properties and prolonged retention at the inflamed sites were evaluated via mucosa adhesion and in vivo imaging system (IVIS) experiments. Finally, the therapeutic efficacy, lesion repair efficiency, and safety of HEP‐Ag‐BSA were comprehensively evaluated based on the dextran sodium sulfate (DSS)‐induced colitis mouse model. It is anticipated that the newly‐designed multi‐functional protein hydrogel may have an important value for clinical transformation with promising application prospects.

## 2 Results and Discussion

### 2.1 Synthesis and Characteristics of the Hydrogels

**Figure 1:**
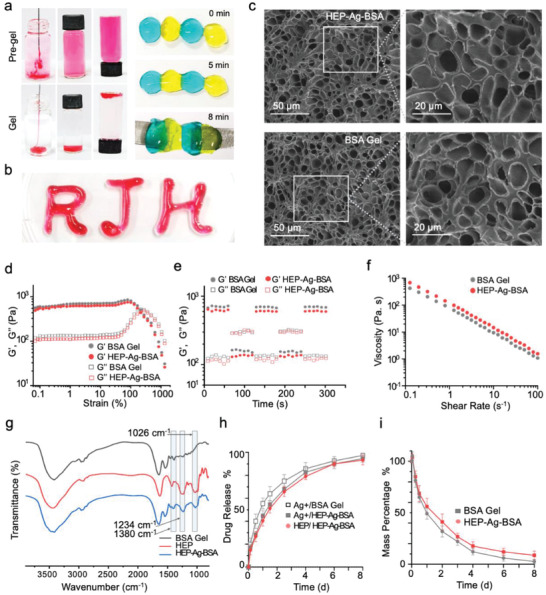
Morphology and characteristics of the hydrogels. a) The gelation process and the self‐healing property of HEP‐Ag‐BSA. b) The injectability of HEP‐Ag‐BSA. c) Morphological observation of the hydrogels via scanning electron microscope (scale bars were 50 and 20 µm, respectively.). d) *G*′ (storage modulus) and *G*″ (loss modulus) of HEP‐Ag‐BSA and BSA Gel in strain sweep measurements. e) Step–strain measurements of HEP‐Ag‐BSA and BSA Gel at 37 °C applying 600% strain for 60 s and 10% strain for 60 s repeatedly. f) Viscosity measurement of HEP‐Ag‐BSA and BSA Gel under varied shear rates. g) Fourier transform infrared spectrum of heparin (HEP), HEP‐Ag‐BSA, and BSA Gel. h) In vitro release of HEP and Ag+ in HEP‐Ag‐BSA and BSA Gel. i) In vitro degradation of HEP‐Ag‐BSA and BSA Gel (*n* = 3, Data were presented as mean ± SD).

The pathogenesis of IBD involves localized disruption of ECM. Intriguingly, the natural protein‐derived protein, BSA, can potentially simulate the 3D microenvironment of ECM, which has been applied in promoting wound healing.^[^ ^29^ ^]^ Thus, in the present study, we constructed a protein hydrogel based on BSA to provide a steady protective microenvironment for tissue repair in IBD. To realize the gelation of BSA, Traut's reagent was applied to replace the —NH_2_ group for the sulfhydryl group, resulting in BSA‐SH. Then, BSA‐SH, heparin, and Ag^+^ were mixed, and the heparin‐functionalized protein hydrogel, HEP‐Ag‐BSA, was obtained via the formation of cross‐linking of Ag^+^ and BSA‐SH, and electrostatic interaction between Ag^+^ and heparin (Scheme 1a). On the other hand, BSA hydrogel (BSA Gel) is formed via coordination between BSA‐SH and Ag^+^. As shown in **Figure** **1**a, the mixture of heparin and BSA‐SH maintained fluidity before adding Ag^+^, and the gelation was swiftly completed within several seconds. Next, the self‐healing property of HEP‐Ag‐BSA was evaluated (Figure 1a; Figure S1a, Supporting Information), four pieces of HEP‐Ag‐BSA dyed in yellow and blue were arranged in a line, and the disappearance of demarcation and infusion of hydrogels was observed after 5 min. Then, an extrusion test was performed, and the letters “RJH” (representing “Ruijin Hospital”) were injected via syringe with a 0.5 mm needle, suggesting that HEP‐Ag‐BSA processed good injectability (Figure 1b; Figure S1b, Supporting Information).

Subsequently, the microstructure of HEP‐Ag‐BSA was analyzed. A homogeneous porous structure could be observed via scanning electron microscope (SEM) in HEP‐Ag‐BSA and BSA Gel (Figure 1c). Rheological measurements were performed to determine the self‐healing characteristics of HEP‐Ag‐BSA and BSA Gel. To demonstrate the linear viscoelastic region of hydrogel, the strain sweep (*γ* = 0.1–1000%) measurements showed that the gel network form and transit to a solution was environmentally at 150% for HEP‐Ag‐BSA and 180% for BSA Gel (Figure 1d). Before high shear rate, HEP‐Ag‐BSA and BSA Gel demonstrated a gel‐forming status with holding storage modulus (*G*′) at ≈600 and ≈700 Pa, respectively; while after high strain at 600% was exerted, the networks of HEP‐Ag‐BSA and BSA Gel were disrupted swiftly with *G*′ dropping to respectively 140 and 160 Pa. In addition, the step‐strain measurements with *γ* = 10% and strain at 600% were used for measuring the recoverability of the two hydrogels under the high strains (Figure 1e), both *G*′ and loss modulus (*G*″) of the two hydrogels could recover ≈100% within a few seconds after the high shear rate ceased, indicating the excellent self‐healing ability of BSA based hydrogel. Besides, both HEP‐Ag‐BSA and BSA Gel displayed shear‐thinning properties, demonstrating that they were suitable biomaterials for injection (Figure 1f).

Next, the structural analysis was performed via Fourier transform infrared (FTIR) spectroscopy, and an absorptive peak at 1380 cm^−1^ for S‐Ag formation, which stood for the core mechanism of gelation, was observed. Enhanced absorption was also observed at 1234 and 1026 cm^−1^, which proved that heparin was successfully introduced into the hydrogel (Figure 1g). In addition, the drug release property of hydrogels was measured in an artificial colonic fluid (ACF, pH = 7.8) environment. As shown in Figure 1h, HEP‐Ag‐BSA released 40.4 ± 4.51% of heparin on day 1, and the release percentage climbed to 93.3 ± 4.16% on day 8. On the other hand, HEP‐Ag‐BSA released 44.7 ± 4.46% of Ag^+^ on day 1, and 94.7 ± 3.06% of Ag^+^ on day 8. To test the degradation property of hydrogels, HEP‐Ag‐BSA and BSA Gel were immersed into ACF and weighted repeatedly at certain time intervals. Figure 1i demonstrates that both the hydrogels degraded fast at the early stage; HEP‐Ag‐BSA degraded almost completely on day 8, while BSA Gel degraded 91.33 ± 4.04%. Therefore, the HEP‐Ag‐BSA could gradually release Ag^+^ and heparin into the colon lumen and reach a relatively high drug concentration early to fulfill the need for local anti‐bacteria, anti‐inflammation, and lesion‐healing.

### 2.2 In Vitro Tests for Biocompatibility of HEP‐Ag‐BSA and Its Impact on Microthrombosis

**Figure 2:**
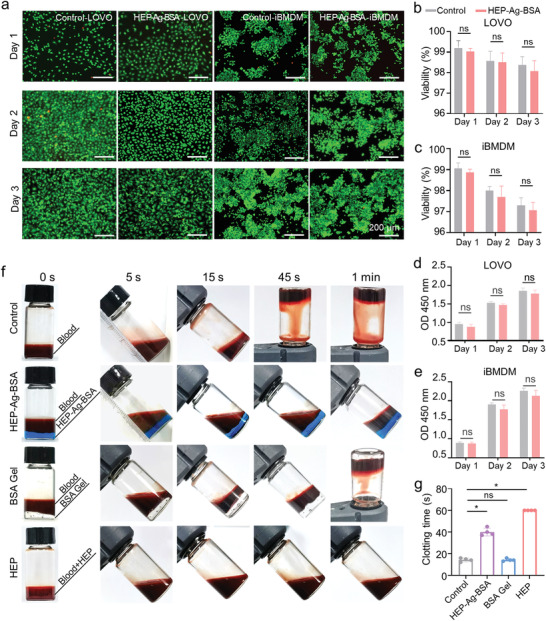
Biocompatibility and in vitro biocompatibility of HEP‐Ag‐BSA and its impact on microthrombosis. a) Fluorescence calcein/PI live/dead staining of LOVOs and iBMDM cells (scale bars were 200 µm). b,c) Viability of different groups via calcein/PI staining. d,e) The proliferation of different groups via the CCK‐8 method (OD for optical density). f) In vitro coagulation test for the hydrogels. g) Clotting time in different groups ((b–e), *n* = 3; (g), *n =* 4; data were presented as mean ± SD; significance between every two groups was determined via Mann–Whitney U‐test).

Reliable safety and therapeutic efficacy are essential preconditions for clinical application. To test the biocompatibility of HEP‐Ag‐BSA, the viability of LOVO cells and immortal bone marrow‐derived macrophages (iBMDM) cells was analyzed via calcein/propidium iodide (calcein/PI) live/dead staining after dropping the leach liquor of HEP‐Ag‐BSA into the cell cultures. As shown in **Figure** **2**a, the cell density of LOVO cells continued to increase, while the viability slightly decreased during the 3‐day incubation. However, there were no statistically significant differences in viability between the control and the HEP‐Ag‐BSA group at the same time point (Figure 2b). Similar results were observed in iBMDM cells (Figure 2a,c). The impact of HEP‐Ag‐BSA on cell proliferation was quantitatively evaluated via a cell counting kit‐8 (CCK‐8) assay. The optical density (OD) at 450 nm of the HEP‐Ag‐BSA and the control group continued to increase without significant intergroup differences in both LOVO and iBMDM cells (Figure 2d,e). The above‐mentioned results revealed that HEP‐Ag‐BSA had good biocompatibility with limiting cytotoxicity against cell proliferation. Furthermore, the biological safety of hydrogels was further confirmed via in vivo experiments using C57BL/6 mice. The pathological analysis of hematoxylin and eosin (H&E) staining slides revealed no significant lesions or organs hemorrhages in vital organs (Figure S2, Supporting Information). These results inferred that HEP‐Ag‐BSA had good biocompatibility, which laid the groundwork for subsequent modeling animal experiments.

The impact of HEP‐Ag‐BSA on microthrombosis was evaluated via in vitro coagulation tests. As shown in Figure 2f, obvious coagulation was observed at 15 s in the control and the BSA Gel group, at ≈1 min in the HEP‐Ag‐BSA group, while the blood sample in the heparin group (HEP group) did not clot. Subsequently, the clotting time was counted. Figure 2g demonstrates that the HEP‐Ag‐BSA and the HEP groups had significantly longer clotting time than the control group (both *p* < 0.05). Based on this, we confirmed that HEP‐Ag‐BSA processed anti‐thrombosis ability, which could modulate the microthrombosis related to inflammation.

### 2.3 In Vitro Antibacterial and Inflammation‐Modulating Properties of the Hydrogels

**Figure 3:**
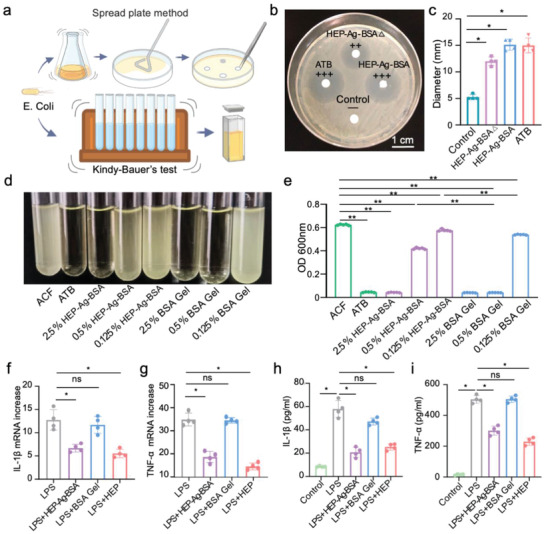
In vitro antibacterial property and inflammation‐modulating properties of the hydrogels. a) Schematic illustration of the Spread plate method and Kindy–Bauer's test. b,c) The antibacterial effect of HEP‐Ag‐BSA against *E. coli* and comparison of the inhibition zone diameters (Δ stands for leach liquid; ATB for antibiotics; scale bar was 1 cm). d,e) Comparison of the antibacterial effect of the hydrogels via Kindy–Bauer's test and the quantitative results (ACF for artificial colonic fluid). f,g) Semi‐quantitative analyses of mRNA levels of pro‐inflammatory interleukin‐1β (IL‐1*β*) and tissue necrosis factor‐α (TNF‐*α*) in iBMDM cells after lipopolysaccharide (LPS) stimulation. h,i) IL‐1*β* and TNF‐*α* levels in iBMDM cell cultures after LPS stimulation ((c), (f–i), *n =* 4; (e), *n =* 5; data were presented as mean ± SD; significance between every two groups was determined via Mann–Whitney U‐test).

Primary and secondary infections frequently occur during the pathogenesis of IBD, which hinders disease recovery. On the other hand, the broad‐spectrum bacteriostatic Ag^+^ is widely applied in developing antibacterial materials.^[^ ^34^ ^]^ As demonstrated in the degradation test, HEP‐Ag‐BSA could continuously release Ag^+^. Based on this, we evaluated the antibacterial properties of the hydrogels via the Spread plate method and Kindy–Bauer's test (**Figure** **3**a). The comparison of diameters of bacteriostatic circles demonstrated a significant anti‐bacterial effect of leach liquid of HEP‐Ag‐BSA, and that of the HEP‐Ag‐BSA hydrogel was even stronger (Figure 3b,c, both *p* < 0.05). The anti‐bacterial effect of the leaching liquid of HEP‐Ag‐BSA and BSA Gel at different concentrations is shown in Figure 3d. The leaching liquid of HEP‐Ag‐BSA and BSA Gel, event at a low concentration of 0.125%, had an anti‐bacterial effect demonstrated by significantly lower OD 600 nm values than the control group with ACF (Figure 3e, both *p* < 0.01). The OD 600 nm value was significantly higher in the tube containing 0.5% HEP‐Ag‐BSA concentration compared to the tube containing 0.5% BSA Gel concentration (Figure 3e, *p* < 0.01), thus indicating that the anti‐bacteria effect of BSA Gel was stronger than HEP‐Ag‐BSA. One possible explanation was that in HEP‐Ag‐BSA, the Ag^+^ ions combined with negatively charged heparin via electrostatic attraction in addition to the S—Ag coordination bond, leading to a relatively lower but still efficient concentration of Ag^+^ for suppressing bacterial proliferation.

On the other hand, existing studies have verified the modulating ability in inflammation reactions of heparin and its derivatives.^[^ ^16^, ^35^, ^36^ ^]^ In addition, the LPS stimulation experiment using the iBMDM cell line was applied to evaluate the anti‐inflammation capability of HEP‐Ag‐BSA.^[^ ^37^ ^]^ Cytokine level of the culture was measured by enzyme‐linked immunosorbent assay (ELISA) kit and mRNA expression level of cells by quantitative real‐time PCR (RT‐qPCR). After 6 h of LPS stimulation, a significant decrease in IL‐1*β* and TNF‐*α* was found in the HEP group and the HEP‐Ag‐BSA group compared with the LPS group (Figure 3f,g, all of *p* < 0.05). Correspondingly, the concentration levels of IL‐1*β* and TNF‐*α* in the cell culture were significantly lower in the HEP and the HEP‐Ag‐BSA groups compared with the LPS group (Figure 3h,i, all of *p* < 0.05). On the other hand, there were no significant differences in IL‐1*β* or TNF‐*α* levels between the LPS and the BSA Gel group in either RT‐qPCR analysis or ELISA tests. From this, we inferred that the HEP‐Ag‐BSA had an anti‐inflammation property mainly due to the composition of heparin, which was consistent with existing reports.^[^ ^38^, ^39^ ^]^

### 2.4 The Lesion‐Adhesive Property of HEP‐Ag‐BSA

**Figure 4:**
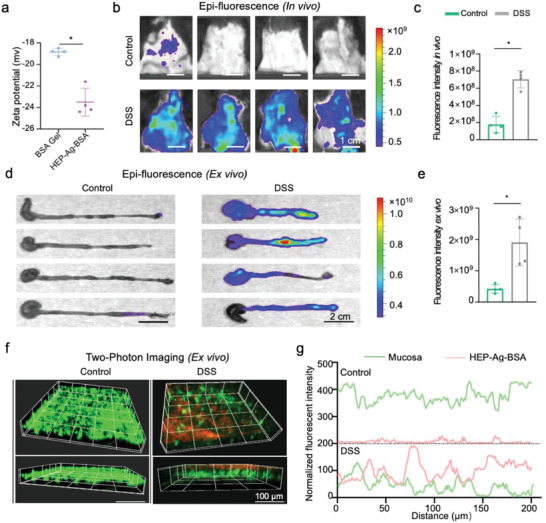
The lesion‐adhesive property of HEP‐Ag‐BSA at the inflamed site. a) Zeta potential of BSA Gel and HEP‐Ag‐BSA. b,c) In vivo fluorescence imaging 6 h after administration of HEP‐Ag‐BSA and comparison of signal intensity (DSS for dextran sodium sulfate; scale bar was 1 cm). d,e) Ex vivo fluorescence imaging of extracted colons and comparison of signal intensity (scale bar was 2 cm). f) Two‐proton imaging for observation of HEP‐Ag‐BSA adhesion at mucosal level (scale bar was 100 µm). g) The relationships between the distribution of fluorescence signal of HEP‐Ag‐BSA and mucosa ((a,c,e), *n =* 4; data were presented as mean ± SD; significance between every two groups was determined via Mann–Whitney U‐test).

IBD features localized lesions in the intestinal tract, where numerous immune cells aggregate and secrete positive‐charged pro‐inflammatory factors, which leads to the reverse of potential difference, with the luminal side being positive.^[^ ^40^ ^]^ On the other side, according to zeta potential measurement, BSA Gel is negatively charged, owing to its protein property, whereas HEP‐Ag‐BSA has even higher negative potential as heparin is a negatively charged macromolecule (**Figure** **4**a). Accordingly, we expected that HEP‐Ag‐BSA might have improved adhesive capability at the lesion site via electrostatic attraction.

To verify the conjecture, in vivo experiments were carried out applying the C57BL/6 mouse model with DSS‐induced colitis featuring high similarity to the anatomical, pathological, and immunological features of the IBD state in humans. To observe the retention of fluorescently labeled HEP‐Ag‐BSA gel (Rhodamine B labeled, red fluorescence) in the colon with DSS‐induced colitis, the IVIS fluorescence imaging was applied to verify the inflammatory adhesion properties. An intensified fluorescence signal was observed in the DSS group (Figure 4b), which was significantly higher compared to the control group (Figure 4c, *p* < 0.05). Also, a similar phenomenon of the extracted colons could be observed via IVIS (Figure 4d), where the DSS group featured a stronger fluorescence signal, representing evident retention of HEP‐Ag‐BSA under the circumstance of colitis (Figure 4e, *p* < 0.05).

Further study for the specific location of adhesion of HEP‐Ag‐BSA in the colon was carried out using multi‐photon‐laser‐scanning‐microscopy (MPLSM) imaging with good tissue penetration. Therefore, we removed the colonic tissues of mice after rectal administration of fluorescently labeled HEP‐Ag‐BSA gel and stained the mucosal layer (wheat germ agglutinin‐FITC staining, green). The distribution and position of the gel to the defective mucosal layer were observed via MPLSM. According to Figure 4f, an evident fluorescence signal of the hydrogel at the mucosal layer was observed in the DSS group but not in the control group. In addition, the fluorescent signal of hydrogel was stronger at the lesion site where the fluorescent signal of mucosa was relatively low (Figure 4g), indicating that HEP‐Ag‐BSA might aggregate at the injured location and deliver therapeutic heparin molecules into deep tissue. At present, a variety of studies applying negatively charged liposomes, gels, and nanoenzyme complexes have succeeded in realizing effective adhesion in the inflamed colon.^[^ ^41^, ^42^, ^43^ ^]^ These shreds of evidence support the long‐term retention of the negatively charged HEP‐Ag‐BSA in the inflammatory site in the present study. In brief, HEP‐Ag‐BSA has a longer retention time in the inflamed colon and a strengthened mucosal penetration at the lesion site, which is beneficial for localized targeting drug delivery.

### 2.5 In Vivo Microthrombosis Modulation Capability and Treatment Safety

**Figure 5:**
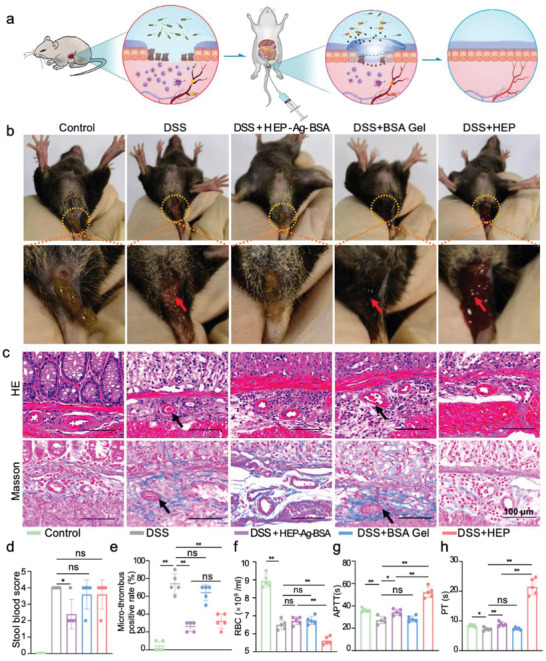
Induction of colitis in mouse and in vivo microthrombosis modulation capability and safety evaluation of HEP‐Ag‐BSA. a) Illustration of HEP‐Ag‐BSA administration in mouse models with DSS‐induced colitis. b) Comparison of severity of hemafecia (red arrows). c) HE staining and Masson's staining of colon tissue sections (black arrows for microthrombi; scale bar was 100 µm). d) Comparison of stool blood score. e) Comparison of the positive rate of micro‐thrombus. f) Evaluation of the severity of bleeding‐related anemia via red blood cell (RBC) counting. g,h) Systemic bleeding risk evaluation via measurement of activated partial thromboplastin time (APTT) and prothrombin time (PT) ((d–h), *n =* 5; data were presented as mean ± SD. Significance between every two groups was determined via Mann–Whitney U‐test).

Based on the above results, HEP‐Ag‐BSA features several advantages, including good injectability, bio‐compatibility, inflamed lesion adhesion, bacteriostasis, and modulating abilities in thrombosis and inflammation. Thus, we promoted in vivo experiments using the DSS‐induced colitis mouse model featuring disturbance of homeostasis of the immune system in the colon to verify the therapeutic effects of HEP‐Ag‐BSA (**Figure** **5**a).^[^ ^44^ ^]^ A thorough evaluation was performed for confirming the thrombolysis efficacy as well as systemic bleeding risk (Figure 5b,c). The DSS group macroscopically featured evident hemafecia while HEP‐Ag‐BSA could improve the condition manifested by a statistically significant decrease in stool bleeding scores (Figure 5b,d, *p* < 0.05). Then, the modulating ability in microthrombosis of the hydrogel was analyzed. The formation of microthrombosis at the submucosa level of the colon in the DSS and the BSA Gel group was obvious via H&E staining, which was more evident in Masson's staining (Figure 5c). However, the microthrombosis was rarely seen in the HEP and the HEP‐Ag‐BSA group, and their micro‐thrombus positivity rate was significantly lower than that of the DSS group (Figure 5e, both *p* < 0.01).

Subsequently, the treatment‐related bleeding risk was evaluated. Important mucosal and sub‐mucosal hemorrhage could be observed in the HEP group via microscopy observation (Figure 5c). The red blood cell (RBC) count was performed to evaluate the severity of anemia, an indicator of systemic hemorrhage. The RBC level was significantly lower in the HEP group than in the HEP‐Ag‐BSA and the DSS group (Figure 5f, both *p* < 0.01), but there were no statistical differences between the HEP‐Ag‐BSA and the DSS group, indicating that systemic heparin dosage aggravated hemorrhage, and the situation was ameliorated in HEP‐Ag‐BSA treatment. Coagulation indicators were also utilized to evaluate systemic bleeding risk. The DSS group had significantly lower activated partial thromboplastin time (APTT) and prothrombin time (PT) than the control group, due to the status of hypercoagulation related to inflammation (Figure 5g,h, *p* < 0.01, *p* < 0.05, respectively.). However, the HEP group had much longer APTT and PT than the control and the HEP‐Ag‐BSA groups (both *p* < 0.01). Heparin has a strong and direct thrombolytic effect, meanwhile, HEP‐Ag‐BSA features controlled release of heparin, realizing an effective treatment effect against localized microthrombosis at the lesion site in inflamed colon and an attenuated impact on coagulation in the systemic circulation. Thus, HEP‐Ag‐BSA demonstrated a definitive therapeutic effect for microthrombosis, which potentially improved tissue repairing by ameliorating micro‐circulation. Furthermore, the safety of HEP‐Ag‐BSA treatment was guaranteed with a relatively low bleeding risk.

### 2.6 HEP‐Ag‐BSA Modulated the Inflammation in DSS‐Induced Colitis

**Figure 6:**
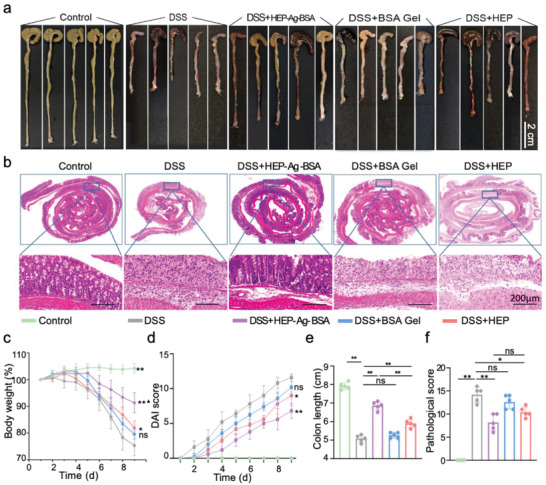
The capability of inflammation modulation of HEP‐Ag‐BSA. a) The comparison of colon length among groups with different treatments (scale bar was 2 cm). b) Pathological evaluation of colon tissue after HE staining (scale bar was 200 µm). c–f) Quantitative analysis of body weight changes, DAI scores, colon length, and pathological score in reflection of inflammation severity among different groups (DAI for disease activity index; (c–f), *n =* 5; data were presented as mean ± SD; significance between every two groups was determined via Mann–Whitney U‐test; ^*^ in (c–d), *p* < 0.05 compared with the DSS group; ▲ in (c), *p* < 0.05 compared with the HEP group).

The DSS‐induced colitis mouse model was also utilized for verifying the therapeutic effects of HEP‐Ag‐BSA in inflammation modulation. Impressively shortened colon length and severe inflammatory injury were displayed in the DSS group (**Figure** **6**a–c). However, the HEP‐Ag‐BSA group featured preserved colon length and alleviated inflammation. Besides, amelioration of body weight loss could be seen in the HEP and the HEP‐Ag‐BSA groups compared with the DSS group (Figure 6c, *p* < 0.05 and *p* < 0.01, respectively.). In addition, the bodyweight of the HEP‐Ag‐BSA group was significantly larger than the HEP group (Figure 6c, *p* < 0.05). By the end of modeling, the HEP and the HEP‐Ag‐BSA group had significantly lower disease activity index (DAI) scores than the DSS group (Figure 6d, *p* < 0.05, *p* < 0.01), which indicated alleviation of colitis‐related symptoms in both groups. Figure 6e demonstrates that the colon length of the HEP and the HEP‐Ag‐BSA groups was significantly longer than that of the DSS group (both *p* < 0.01). The HEP‐Ag‐BSA group had an even significantly longer colon length than the HEP group (*p* < 0.01). In addition, the HEP‐Ag‐BSA and the HEP group had significantly lower pathological scores than the DSS group (Figure 6f, *p* < 0.01 and *p* < 0.05, respectively. ).

The aforementioned results demonstrated that HEP‐Ag‐BSA had an excellent therapeutic effect against DSS‐induced colitis and outperformed the traditional heparin therapy, which might be due to the protein hydrogel platform that enabled a longer retention time and better penetration ability at inflamed mucosa and that this biomaterial could alleviate inflammatory tissue damage by providing an ECM‐mimicking micro‐environment with increased drug concentration, which allowed a stronger therapeutic effect.

### 2.7 The Mechanism of Inflammation Modulation and Tissue Repair of HEP‐Ag‐BSA

**Figure 7:**
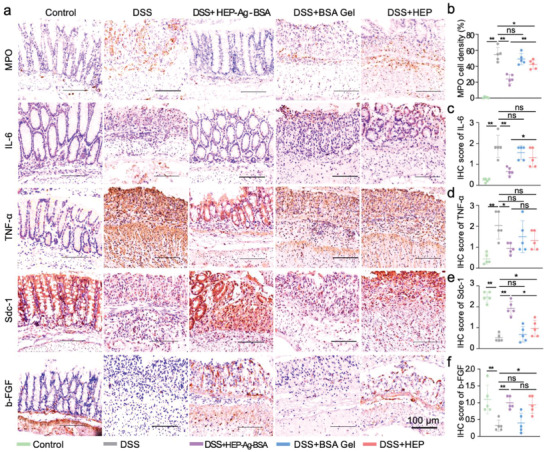
Immunohistological analysis of factors contributing to the pathogenesis of IBD. a) Immunohistochemistry (IHC) coloring of pro‐inflammatory factors and repairing factors in intestinal sections (scale bar was 100 µm). b) Comparison of myeloperoxidase (MPO) positive cell density. c–f) IHC scores of IL‐6, TNF‐*α*, Syndecan‐1 (Sdc‐1), and basic fibroblast growth factor (b‐FGF) in different groups ((b–f), *n =* 5; data were presented as mean ± SD; significance between every two groups was determined via Mann–Whitney U‐test).

To further explore the mode of action of HEP‐Ag‐BSA, the immunohistochemistry (IHC) approach was employed to analyze classical pro‐inflammatory factors and repairing factors (**Figure** **7**a). Three inflammation‐related cytokines, myeloperoxidase (MPO), IL‐6, and TNF‐*α*, and two tissue repair factors, Syndecan‐1 (Sdc‐1) and basic fibroblast growth factor (b‐FGF) were chosen for IHC staining. A great density of MPO positive immune cells and a large region of ECM intensely stained with IL‐6 and TNF‐*α* at the mucosal and submucosal level of colon tissue were seen in the DSS group, while the percentage of MPO positive cells and the intensity of staining of IL‐6 and TNF‐*α* were decreased evidently in the HEP and the HEP‐Ag‐BSA group. As demonstrated in Figure 7b–d, the MPO positive cell percentage, IHC scores of IL‐6 and TNF‐*α* in the HEP‐Ag‐BSA group were significantly lower than those of the DSS group (*p* < 0.01, *p* < 0.01, *p* < 0.05, respectively.), and the HEP group had a lower MPO positive cell density compared with the DSS group (*p* < 0.05). Figures S3,S4, Supporting Information measure mRNA expressions and serum concentration of TNF‐*α* and IL‐1*β*. According to the semi‐quantitative analyses, the mRNA level of TNF‐*α* and IL‐1*β* were all significantly lower in the HEP‐Ag‐BSA and the HEP group than in the DSS group (*p* < 0.01, *p* < 0.01, *p* < 0.01, *p* < 0.05), and there existed a significantly decrease in serum TNF‐*α* and IL‐1*β* in the HEP‐Ag‐BSA group compared to the DSS group (both *p* < 0.01). On the other hand, Figure 7a shows intense staining of both Sdc‐1 and b‐FGF at the mucosal and sub‐mucosal level in the HEP‐Ag‐BSA group, while the Sdc‐1 staining in the HEP group was also evident. The IHC scores of Sdc‐1 and b‐FGF were significantly higher in the HEP‐Ag‐BSA and the HEP group than in the DSS group (Figure 7e,f, *p* < 0.01, *p* < 0.05, *p* < 0.01, *p* < 0.05, respectively.). The IHC scores of Sdc‐1 were significantly higher in the HEP‐Ag‐BSA group than in the HEP group (Figure 7e, *p* < 0.05).

In short, HEP‐Ag‐BSA had an evident therapeutic effect on IBD. On one hand, it could exert an inflammation‐modulating effect via suppressing the expression of pro‐inflammatory factors, as reported in available studies.^[^ ^45^, ^46^ ^]^ It is worth mentioning that, IL‐6 acts as a mediator in microthrombosis in IBD by enhancing platelet activation and promoting platelet‐leukocyte aggregation.^[^ ^47^ ^]^ HEP‐Ag‐BSA is effective in decreasing IL‐6 levels at inflammatory sites, which renders it suitable for controlling inflammation‐related microthrombosis. On the other hand, HEP‐Ag‐BSA can improve mucosal healing by boosting certain protective factors. For example, Sdc‐1 is physically expressed on the cellular membrane, maintaining the mucosal barrier of the gastrointestinal tract.^[^ ^48^ ^]^ Heparin is an analog of Sdc1 side chain and may alleviate tissue damage as a substitute for Sdc‐1.^[^ ^49^ ^]^ B‐FGF, an epithelial mitogen stimulating angiogenesis, and ECM remodeling, contains specific heparin‐binding sites of which heparin can restore the high‐affinity receptor and improves mucosal recovery.^[^ ^50^, ^51^ ^]^ Furthermore, hydrogel is a versatile carrier for water‐soluble drugs and can mimic the biological tissue structure and properties.^[^ ^52^, ^53^, ^54^ ^]^ BSA deviated hydrogel can serve as a 3D scaffold and assist cell culture. In the present study, the BSA‐based hydrogel can patch the location of mucosal injury, function as a barrier against pathogens from the gut lumen, and provide a versatile environment for tissue repairing and disease recovery.

## 3 Conclusions

In summary, to balance microthrombosis and inflammation in IBD, this study presented a heparin‐functionalized protein hydrogel based on silver ion mediated coordination with thiolated BSA and electronic interaction with heparin for balancing inflammation and microthrombosis in the pathogenesis of IBD. The dynamic S‐Ag coordination equipped HEP‐Ag‐BSA with gradual degrading, self‐healing, injectable, and antibacterial properties, enabling it to be injected per rectum as an enema to realize localized drug administration. Furthermore, it could anchor on inflamed mucosa with prolonged retention via electrostatic action, thus further improving bioavailability. Also, HEP‐Ag‐BSA continuously released heparin, effectively balancing inflammation and microthrombosis, preventing secondary degeneration related to ischemia and hypoxia, and promoting disease recovery with guaranteed biocompatibility and safety. However, the study has its limitations in that the animal model represents particularly an acute phase of inflammation, thus, the therapeutic effect of HEP‐Ag‐BSA should be further analyzed in other situations before clinical applications. Nonetheless, the design of this multifaceted protein hydrogel platform is expected to open up a new field in the comprehensive management of IBD.

## 4 Experimental Section

### Preparation of the Protein Hydrogels

First, 5 g of BSA (Bidepharm, CAS: 9048‐46‐8, China) was dissolved into a 200 mL working buffer composed of 0.05 m phosphate‐buffered saline (PBS) and 0.005 m ethylene diamine tetra‐acetic acid at 0 °C. Second, 520 mg 2‐iminothiolane hydrochloride (Aladdin, CAS: 4781‐83‐3, China) was dissolved into a 26 mL working buffer and added drop‐by‐drop to the BSA solution. The solution was stirred continuously for 4 h at 0 °C for reaction. Then, a dialyze system (MWCO 3500, Spectrum) with deionized water (5 < pH < 6) was established for the purification of the prepared BSA‐SH solution for 2 days. After that, the dialyzed solution was lyophilized in a lyophilizer (SCIENTZ‐18N, China) for 2 days and then stored at −20 °C.

Lyophilized BSA‐SH was dissolved in deionized water to form a 35% w/w pre‐gel. The solution was vortexed for 30 s and then stirred gently using a small magnetic stir overnight at 4 °C to reach a homogenous status. To prepare BSA Gel, 0.2 m silver nitrate (AgNO3) solution was prepared and added to the solution drop‐wise with quick stirring and finally reach a concentration at 0.015 m. To prepare HEP‐Ag‐BSA, heparin sodium salt (Aladdin, CAS: 9041‐08‐1, China) was dissolved in deionized water and mixed with BSA pre‐gel to reach a concentration of 3.5 mg mL^−1^. After that, AgNO_3_ solution was added and finally reached the same concentration as mentioned above.

### Morphology Observation

HEP‐Ag‐BSA and BSA Gel were freeze‐dried with liquid nitrogen and then fixed on a round plate of 10 mm diameter using carbon tape. Gold was sprayed on the surface of the frozen hydrogels in 30 s to form a layer. The samples were then observed via SEM (Hitachi, S‐4800).

### FTIR Analysis

HEP‐Ag‐BSA and BSA Gel were freeze‐dried with liquid nitrogen. The FTIR (Nicolet 6700; Thermo Scientific, USA) was used to analyze the chemical structure of the hydrogels. Powdered heparin sodium salt was also examined by FTIR for comparison of chemical structures changes.

### Zeta Potential Measurement

The zeta potential of the hydrogels was measured by dynamic light scattering (DLS) equipped with ZetaPlus Zeta Potential Analyzer. A block of 100 µL HEP‐Ag‐BSA or BSA Gel was put into 2 mL distilled water and the tube was placed on a rotary shaker for 5 min. Then the zeta potential was measured via DLS and repeated four times.

### Rheology Analysis

To determine the rheological characteristics of hydrogels, 400 µL HEP‐Ag‐BSA or BSA Gel were placed on a 4 cm heating horizontal plate at 37 °C for each test. The oscillation mode was selected. The frequency was set at 10 rad s^−1^. Each sample underwent the following process: 10% strain for 60 s, 600% strain for 60 s, 10% strain for 60 s, 600% strain for 60 s, and 5% strain for 60 s.

### In Vitro Swelling and Degradation Tests

An ACF (pH = 7.8) was prepared by mixing 5.59 g Na_2_HPO_4_ and 0.41 g KH_2_PO_4_ into 1 L deionized water to mimic a colonic environment for in vitro study. To determine the swelling and degradation character of hydrogels, 1 mL HEP‐Ag‐BSA and BSA Gel were separately placed in 5 mL ACF at 37 °C. At each time point, the dry weight was measured and the swelling and degradation rate of HEP‐Ag‐BSA and BSA Gel were calculated.

### In Vitro Release Experiments

The in vitro drug release experiment of heparin and Ag^+^ was realized in ACF. A piece of 500 µL of hydrogel was placed in 2 mL ACF at 37 °C. At each time point, 2 mL ACF was taken out and fresh ACF of equivalent volume was added back into the samples. The ACF extract was used for the measurement of concentrations. The concentrations of released heparin were measured via a colorimetric method applying Azure A chloride according to standard protocol (Rebio, China). The concentrations of released Ag^+^ ions were measured via high‐performance liquid chromatography (Waters, H‐Class, US).

### Cell Culture

Mouse iBMDM and human colon carcinoma cells (LOVO) were obtained via commercial approaches and used for in vitro experiments. LOVO cells were cultured in Dulbecco's modified Eagle's medium (Gibco, USA) supplemented with 10% fetal bovine serum (FBS, Gibco, USA). IBMDM cells were cultured in RPMI 1640 culture (Gibco, USA) containing 10% FBS. LOVO and iBMDM were cultured in an incubator with a constant temperature of 37 °C with 5% CO_2_.

### In Vitro Biocompatibility Tests

As for in vitro experiment, both iBMDM and LOVO cells were seeded in 96‐well plates, and leach liquor of HEP‐Ag‐BSA was added to the cultures. After incubation for 1, 2, and 3 days, CCK‐8 (Beyotime, China) was used to determine the viability percentage using a microplate reader, and calcein/PI cell live/dead staining kit (Beyotime, China) was used to observe the live and dead cells via a fluorescent microscope and the percentage of live cells were calculated. The procedure of the CCK‐8 assay and calcein/PI staining were carried out according to the standard protocol.

### In Vitro Coagulation Test

Whole blood sample from SD rats was applied for in vitro coagulation test. Sample bottles loaded with 500 µL HEP‐Ag‐BSA concentration, BSA Gel, or 10 UI heparin were injected with 1 mL blood sample separately. The bottles were placed at an angle of 45° every 3 s to detect coagulation. The clotting time was recorded.

### In Vitro Anti‐Inflammation Property

IBMDM cells were cultured in 6‐well plates. LPS at 1 µg mL^−1^ was used to stimulate the cells. Leach liquor of HEP‐Ag‐BSA was added to cell culture to test its anti‐inflammation property. After 6 h of stimulation, the total RNA of cells was extracted using Trizol (Takara, Japan). After that, a cDNA Synthesis Kit (Yeasen, China) was applied to transcribe extracted RNA into cDNA. Then, RT‐qPCR was performed for semi‐quantification of the expression level of certain factors related to inflammation.

Here listed the primer sequences:

IL‐1*β*: Forward TTCAGGCAGGCAGTATCACTC

Reverse GAAGGTCCACGGGAAAGACAC

IL‐6: Forward CTGCAAGAGACTTCCATCCAG

Reverse AGTGGTATAGACAGGTCTGTTGG

*β*‐actin: Forward CGTTGACATCCGTAAAGACC

Reverse TAGGAGCCAGAGCAGTAATC

TNF‐*α* 1: Forward CAGGCGGTGCCTATGTCTC

Reverse CGATCACCCCGAAGTTCAGTAG

### In Vitro Anti‐Bacterial Test

The anti‐bacterial property of hydrogels was evaluated by Kirby–Bauer (KB) inhibition zone assay against *Escherichia coli* and a comparison of OD of *E. coli* culture. *E. coli* were cultured in Luria‐Bertani (LB) medium at 37 °C for 24 h in a shaking incubator. In the KB inhibition zone assay, *E. coli* were evenly smeared on the agar surface of the plate. Then, round filter papers with a diameter of 6 mm containing 20 µL of leach liquor of HEP‐Ag‐BSA/ 20 µL of antibiotics (100 U mL^−1^ penicillin and 100 µg mL^−1^ streptomycin)/ACF and a round piece of HEP‐Ag‐BSA of 6 mm in diameter were placed on the ager surface separately. The diameters of inhibition zones were measured after 12 h of co‐incubation at 37 °C. The procedures were repeated four times. In the OD measurement, the LB culture of *E. coli* was divided evenly into eight tubes. Antibiotics, leach liquor of HEP‐Ag‐BSA, or BSA Gel at different concentrations were added separately. After another incubation of 12 h, the 600 nm OD scores were measured by a microplate reader 5 times.

### Animal Subjects

The experiment was carried out with the approval of the Institutional Animal Care & Use Committee (IACUC) of Shanghai Jiao Tong University School of Medicine (Approval No. B‐2021‐009). 8 week‐old male C57BL/6 mice (Lingchang, China) were purchased and raised in a specific‐pathogen‐free (SPF) room with a constant temperature of 20 °C, in a 12 h day/12 h night life cycle. Before modeling, all the animals were roomed in the SPF room for 1 week, having free access to distilled water and standard mouse chow.

### In Vivo Biocompatibility Tests

C57BL/6 mice were utilized for in vivo tests for the evaluation of biocompatibility. HEP‐Ag‐BSA or BSA Gel was injected par rectum daily for 8 days, and saline was used in the control group. Then, all the animals were sacrificed and vital organs (lung, liver, kidney, spleen, and heart) were extracted and pathological analyses after HE staining were performed to verify treatment‐related inflammation and hemorrhage.

### DSS‐Induced Colitis Mice Modeling and Treatment Procedures

All the mice were randomly distributed into the control group, the DSS group, the HEP‐Ag‐BSA group, the BSA Gel, and the HEP group. The control group was given distilled water while the rest were given 3% w/v DSS water (DSS, Millipore, US) to induce colitis. Fresh DSS drinking water was prepared and old ones were replaced every 2 days. From the date of DSS administration, the HEP‐Ag‐BSA and the BSA Gel group were given enemas of 200 µL HEP‐Ag‐BSA (heparin: 1000 U kg^−1^) or BSA Gel per rectum once daily using an elastic enema tube under anesthesia, while the HEP group received heparin intra‐peritoneally at 400 IU kg^−1^ twice daily.

During modeling, body weight change, stool consistency, and the occurrence of hemafecia were noted, and the stool was collected. DAI score was used to evaluate the severity of symptoms according to weight loss, stool consistency, and occult or gross bleeding. All the animals were sacrificed after 8 days of DSS administration. Blood was extracted after anesthesia and the colon tissue was collected after sacrifice.

### In Vivo Imaging System

C57BL/6 mice with DSS‐induced colitis and the control group were intra‐rectally given 200 µL of Rodamine‐labeled HEP‐Ag‐BSA. The IVIS imaging of mice was carried out 6 h after administration of HEP‐Ag‐BSA, and the fluorescence intensity was measured. Then, all the mice were sacrificed and the colons were extracted for IVIS imaging. The images and the fluorescence signals were recorded for further analysis.

### Evaluation of Mucus Adhesion Properties

The colon tissue was excised after IVIS examination and cut into 1.5 cm loops. The colon tissue was put under examination after mucosal staining (wheat germ agglutinin‐FITC staining, green). The tissue was embedded and fixed to a coverslip suction head apparatus (Olympus, Japan). A vacuum pump was applied to guarantee the continuous suction of tissue during imaging. FVMPE‐RS (Olympus, Japan) a MPLSM was applied to obtain two‐photon imaging using 900 nm laser for excitation, 35 mW laser power, 10× water immersion objective lens (Olympus, Japan, 0.95 numerical aperture (NA)). Optical sections were collected every 1 µm in the *z*‐axis direction, and the final imaging was formed via the z‐stack projections. The Image J software was utilized to observe and measure the mucosal coverage area with red fluorescence.

### HE or Masson's Staining and Immunohistological Staining

Samples were harvested and fixed in 4% paraformaldehyde overnight then dehydrated and embedded in paraffin blocks. After that, 5 µm histological sections were cut at the center of the specimens. Finally, the sections were stained with H&E or Masson's trichrome. For IHC staining, the paraffin slices were deparaffinized using 3% hydrogen peroxide in 10 min, blocked by blocking buffer for an hour, and then incubated in primary antibody for 12 h at 4 °C, including MPO at 1:300, IL‐6 at 1:100, TNF‐*α* at 1:100, Syndecan‐1 at 1:2000, and b‐FGF at 1:1000. The slices were immersed in the dilution buffer containing secondary antibody for 1 h at 20 °C and then rinsed with PBS 3 times. The DAB (Agilent Technologies, US) was added for the color reaction according to the standard protocol.

An optical microscope was used for observation of the prepared sections. The KFBIO viewer (Ver 1, China) was applied for recording the images of tissue slices and scoring. The observation and IHC scoring of tissue was performed by two investigators separately. Only the membranes of epithelial cells, glandular cells in the epithelium, and sub‐epithelium cells with positive staining were scored. The percentage of positive cells was determined by the number of positive cells versus the total cells number in 10 randomly selected high‐power fields (×400) in each sample. The IHC score was determined by multiplication of two coefficients: the staining intensity score (0 for negative, 0.5 for trace, 1 for light, 2 for moderate, and 3 for intense) and the percentage of positively stained cells (0 for negative, 0.1 for under 25%, 0.4 for 26–50%, 0.6 for 51–75%, and 0.9 for 76–100%).

### Anticoagulation Test of Serum

After induction of the DSS colitis model, whole blood samples of mice were extracted under anesthesia. For anticoagulation tests, blood samples were collected in an anticoagulant tube containing sodium citrate. Then the samples were centrifuged at 3000 rpm for 15 min within 1 h. The supernatant was collected and tested using an automatic coagulation analyzer (Rayto, China).

### In Vivo Anti‐Inflammation Property

Mice were sacrificed after DSS modeling, blood samples were stored at 4 °C overnight and then centrifuged at 3000 rpm for 10 min. The supernatant was collected for a further test of inflammation‐related IL‐1*β* and TNF‐*α* according to standard protocols of ELISA Kits (Thermo Fisher, Austria). Mouse colon was extracted and total RNA of mouse colon tissue was obtained and RT‐qPCR analysis was carried out in the same way as mentioned above.

### Statistical Analysis

Data analysis was realized using Statistical Package for the Social Sciences version 22.0 (SPSS, IBM, Chicago, US) and GraphPad Prism 8.0 (GraphPad, La Jolla, CA, USA). Quantitative data were described by the means with standard deviation (SD). Categorical variables were expressed in numbers with percentages. The Mann–Whitney U‐test and one‐way analysis of variance were used for calculating the statistical significances. *p* value under 0.05 was considered of statistical significance (ns not statistically significant, ^*^ *p* < 0.05, ^**^ *p* < 0.01).

## Conflict of Interest

The authors declare no conflict of interest.

## Supporting information

Supporting Information

## Data Availability

Research data are not shared.

## References

[1] H. Nakase , M. Uchino , S. Shinzaki , M. Matsuura , K. Matsuoka , T. Kobayashi , M. Saruta , F. Hirai , K. Hata , S. Hiraoka , M. Esaki , K. Sugimoto , T. Fuji , K. Watanabe , S. Nakamura , N. Inoue , T. Itoh , M. Naganuma , T. Hisamatsu , M. Watanabe , H. Miwa , N. Enomoto , T. Shimosegawa , K. Koike , J. Gastroenterol. 2021, 56, 489. 33885977 10.1007/s00535-021-01784-1 PMC8137635

[2] M. Hayat , R. A. Ariëns , P. Moayyedi , P. J. Grant , S. O'Mahony , Eur. J. Gastroenterol. Hepatol. 2002, 14, 249. 11953689 10.1097/00042737-200203000-00008

[3] T. Li , C. Wang , Y. Liu , B. Li , W. Zhang , L. Wang , M. Yu , X. Zhao , J. Du , J. Zhang , Z. Dong , T. Jiang , R. Xie , R. Ma , S. Fang , J. Zhou , J. Shi , J. Crohn's Colitis. 2020, 10, 240. 10.1093/ecco-jcc/jjz132 31325355

[4] M. Mussbacher , M. Salzmann , C. Brostjan , B. Hoesel , C. Schoergenhofer , H. Datler , P. Hohensinner , J. Basílio , P. Petzelbauer , A. Assinger , J. A. Schmid , Front. Immunol. 2019, 10, 85. 30778349 10.3389/fimmu.2019.00085 PMC6369217

[5] M. Witkowski , U. Landmesser , U. Rauch , Trends. Cardiovasc. Med. 2016, 26, 297. 26877187 10.1016/j.tcm.2015.12.001

[6] S. Danese , A. Papa , S. Saibeni , A. Repici , A. Malesci , M. Vecchi , Am. J. Gastroenterol. 2007, 102, 174. 17100967 10.1111/j.1572-0241.2006.00943.x

[7] J. R. Korzenik , Inflamm. Bowel Dis. 1997, 3, 87. 23282750

[8] A. J. Wakefield , A. M. Sawyerr , A. P. Dhillon , R. M. Pittilo , P. M. Rowles , A. A. Lewis , R. E. Pounder , Lancet. 1989, 2, 1057. 2572794 10.1016/s0140-6736(89)91078-7

[9] E. S. Brown , Ann. N. Y. Acad. Sci. 2009, 1179, 41.19906231

[10] V. Linares , V. Alonso , J. L. Domingo , Expert Opin. Drug Saf. 2011, 10, 253. 21219240 10.1517/14740338.2011.529898

[11] J. Torres , S. Mehandru , J. F. Colombel , L. Peyrin‐Biroulet , Lancet. 2017, 389, 1741. 27914655 10.1016/S0140-6736(16)31711-1

[12] M. Fernández‐Ruiz , J. M. Aguado , Expert Rev. Anti Infect. Ther. 2018, 16, 939. 30388900 10.1080/14787210.2018.1544490

[13] P. Pavlidis , I. Bjarnason , Curr. Pharm. Des. 2015, 21, 5089. 26369683 10.2174/1381612821666150915110058

[14] M. Cattaneo , J. Thromb. Haemost. 2015, 13, S10. 26149010 10.1111/jth.12952

[15] E. Mantuano , P. Azmoon , C. Brifault , M. A. Banki , A. S. Gilder , W. M. Campana , S. L. Gonias , Blood 2017, 130, 1364. 28684538 10.1182/blood-2017-04-780205 PMC5600142

[16] E. Young , Thromb. Res. 2008, 122, 743. 17727922 10.1016/j.thromres.2006.10.026

[17] X. Li , X. Ma , Br. J. Haematol. 2017,179, 389. 28832958 10.1111/bjh.14885

[18] N. Chande , J. W. D. McDonald , J. K. MacDonald , J. J. Wang , Cochrane Database Syst. Rev. 2010, 10.1002/14651858.CD006774.pub3.

[19] Q. Y. Lean , N. Gueven , R. D. Eri , R. Bhatia , S. S. Sohal , N. Stewart , G. M. Peterson , R. P. Patel , Expert Rev. Clin. Pharmacol. 2015, 8, 795. 26308504 10.1586/17512433.2015.1082425

[20] H. F. Cui , X. L. Jiang , World J. Gastroenterol. 1999, 5, 448. 11819488 10.3748/wjg.v5.i5.448 PMC4688620

[21] Y. Meissner , Y. Pellequer , A. Lamprecht , Int. J. Pharm. 2006, 316, 138. 16675176 10.1016/j.ijpharm.2006.01.032

[22] T. Yazeji , B. Moulari , A. Beduneau , V. Stein , D. Dietrich , Y. Pellequer , A. Lamprecht , Drug Delivery 2017, 24, 811. 28509629 10.1080/10717544.2017.1324530 PMC8240985

[23] S. K. Ramadass , S. Perumal , S. L. Jabaris , B. Madhan , Eur. J. Pharm. Sci. 2013, 23, 104. 10.1016/j.ejps.2012.10.015 23137838

[24] J. Xu , M. Tam , S. Samaei , S. Lerouge , J. Barralet , M. M. Stevenson , M. Cerruti , Acta Biomater. 2017, 48, 247. 27769943 10.1016/j.actbio.2016.10.026

[25] L. Zhao , F. Wang , Z. Cai , Q. Zhou , B. Chen , C. Zhang , H. Liu , L. Hong , T. Zhang , J. Zhong , W. Cui , Z. Wang , Chem. Eng. J. 2021, 424, 130464.

[26] C. Liu , X. Xu , W. Cui , H. Zhang , Engineered Regeneration 2021, 2, 105.

[27] L. Cai , D. Xu , H. Chen , L. Wang , Y. Zhao , Engineered Regeneration 2021, 2, 109.

[28] Z. Cai , Q. Saiding , L. Cheng , L. Zhang , Z. Wang , F. Wang , X. Chen , G. Chen , L. Deng , W. Cui , Bioact. Mater. 2021, 6, 4506. 34027237 10.1016/j.bioactmat.2021.04.039 PMC8134719

[29] L. Cheng , Z. Cai , T. Ye , X. Yu , Z. Chen , Y. Yan , J. Qi , L. Wang , Z. Liu , W. Cui , L. Deng , Adv. Funct. Mater. 2020, 30, 2001196.

[30] B. Tirosh , N. Khatib , Y. Barenholz , A. Nissan , A. Rubinstein , Mol. Pharm. 2009, 6, 1083. 19603812 10.1021/mp9000926

[31] M. S. Lepanto , L. Rosa , R. Paesano , P. Valenti , A. Cutone , Molecules. 2019, 24, 1323. 10.3390/molecules24071323 PMC6480387 30987256

[32] M. R. Scheenstra , R. M. van Harten , E. J. A. Veldhuizen , H. P. Haagsman , M. Coorens , Front. Immunol. 2020, 11, 1137. 32582207 10.3389/fimmu.2020.01137 PMC7296178

[33] T. L. Lopez‐Silva , D. G. Leach , A. Azares , I. C. Li , D. G. Woodside , J. D. Hartgerink , Biomaterials. 2020, 231, 119667. 31855625 10.1016/j.biomaterials.2019.119667 PMC7049098

[34] K. S. Siddiqi , A. Husen , R. A. K. Rao , J. Nanobiotechnology 2018, 16, 14. 29452593 10.1186/s12951-018-0334-5 PMC5815253

[35] X. F. Wang , A. M. Li , J. Li , S. Y. Lin , C. D. Chen , Y. L. Zhou , X. Wang , C. L. Chen , S. D. Liu , Y. Chen , PLoS One. 2013, 8, e66397. 23874391 10.1371/journal.pone.0066397 PMC3715511

[36] S. Chen , Y. He , Z. Hu , S. Lu , X. Yin , X. Ma , C. Lv , G. Jin , J. Histochem. Cytochem. 2017, 65, 241. 28170292 10.1369/0022155417692536 PMC5407566

[37] A. Tanaka , J. To , B. O'Brien , S. Donnelly , M. Lund , BMC Immunol. 2017, 18, 43. 28974200 10.1186/s12865-017-0223-y PMC5627409

[38] S. Anastase‐Ravion , C. Blondin , B. Cholley , N. Haeffner‐Cavaillon , J. J. Castellot , D. Letourneur , J. Biomed. Mater. Res., Part A 2003, 66, 376. 10.1002/jbm.a.10604 12889008

[39] L. Chimenti , M. Camprubí‐Rimblas , R. Guillamat‐Prats , M. N. Gomez , J. Tijero , L. Blanch , A. Artigas , Thromb. Haemost. 2017, 117, 2125. 29202212 10.1160/TH17-05-0347 PMC6328369

[40] C. J. Edmonds , Gut 1970, 11, 867. 4395004 10.1136/gut.11.10.867 PMC1553147

[41] T. T. Jubeh , Y. Barenholz , A. Rubinstein , Pharm. Res. 2004, 21, 447. 15070095 10.1023/B:PHAM.0000019298.29561.cd

[42] S. Zhang , J. Ermann , M. D. Succi , A. Zhou , M. J. Hamilton , B. Cao , J. R. Korzenik , J. N. Glickman , P. K. Vemula , L. H. Glimcher , G. Traverso , R. Langer , J. M. Karp , Sci. Transl. Med. 2015, 12, 300ra128. 10.1126/scitranslmed.aaa5657 PMC4825054 26268315

[43] S. Zhao , Y. Li , Q. Liu , S. Li , Y. Cheng , C. Cheng , Z. Sun , Y. Du , C. J. Butch , H. Wei , Adv. Funct. Mater. 2020, 30, 2004692.

[44] B. Chassaing , J. D. Aitken , M. Malleshappa , M. Vijay‐Kumar , Curr. Protoc. Immunol. 2014, 104, 15.25.1. 24510619 10.1002/0471142735.im1525s104 PMC3980572

[45] X. Li , Y. Liu , L. Wang , Z. Li , X. Ma , Immunobiology 2015, 220, 1197. 10.1016/j.imbio.2014.10.008 25454806

[46] A. Salas , M. Sans , A. Soriano , J. C. Reverter , D. C. Anderson , J. M. Piqué , J. Panés , Gut 2000, 47, 88. 10861269 10.1136/gut.47.1.88 PMC1727984

[47] S. L. Yan , J. Russell , D. N. Granger , Inflamm. Bowel Dis. 2014, 20, 353. 24390064 10.1097/01.MIB.0000440614.83703.84 PMC3947085

[48] A. N. Alexopoulou , H. A. Multhaupt , J. R. Couchman , Int. J. Biochem. Cell Biol. 2007, 39, 505. 17097330 10.1016/j.biocel.2006.10.014

[49] M. Floer , M. Götte , M. K. Wild , J. Heidemann , E. S. Gassar , W. Domschke , L. Kiesel , A. Luegering , T. Kucharzik , Am. J. Pathol. 2010, 176, 146. 20008145 10.2353/ajpath.2010.080639 PMC2797877

[50] N. P. Michell , P. Lalor , M. J. Langman , Eur. J. Gastroenterol. Hepatol. 2001, 13, 449. 11338079 10.1097/00042737-200104000-00026

[51] R. Day , A. Forbes , Lancet 1999, 354, 62. 10406379 10.1016/S0140-6736(98)09267-8

[52] S. Sharma , S. Tiwari , Int. J. Biol. Macromol. 2020, 162, 737. 32553961 10.1016/j.ijbiomac.2020.06.110

[53] L. T. Saldin , M. C. Cramer , S. S. Velankar , L. J. White , S. F. Badylak , Acta Biomater. 2017, 49, 1. 27915024 10.1016/j.actbio.2016.11.068 PMC5253110

[54] S. Naahidi , M. Jafari , M. Logan , Y. Wang , Y. Yuan , H. Bae , B. Dixon , P. Chen , Biotechnol. Adv. 2017, 35, 530. 28558979 10.1016/j.biotechadv.2017.05.006

