# Supplementary material for: Balancing Microthrombosis and Inflammation via Injectable Protein Hydrogel for Inflammatory Bowel Disease
Source: Adv Sci (Weinh). 2022 May 7;9(20):2200281. doi: 10.1002/advs.202200281 (PMC9284187; doi:10.1002/advs.202200281)
Supplement: Supplementary file 1 — Supporting Information [file ADVS-9-2200281-s001.pdf]

Supporting Information

Balancing Microthrombosis and Inflammation via Injectable Protein Hydrogel for Inflammatory Bowel Disease

Liwen Hong, Gaoxian Chen, Zhengwei Cai, Hua Liu, Chen Zhang, Fei Wang, Zeyu Xiao, Jie Zhong, Lei Wang, Zhengting Wang*, and Wenguo Cui**

Dr. L. Hong, Dr. H. Liu, Dr. C. Zhang, Prof. J. Zhong, Dr. L. Wang, and Dr. Z. Wang
Department of Gastroenterology, Ruijin Hospital, Shanghai Jiao Tong University School of Medicine, 197 Ruijin 2nd Road, Shanghai 200025, P. R. China.
E-mail: wl10779@rjh.com.cn (L. Wang), zhengtingwang@shsmu.edu.cn (Z. Wang)

Dr. L. Hong, Dr. Z. Cai, Dr. F. Wang, and Prof. W. Cui
Department of Orthopaedics, Shanghai Key Laboratory for Prevention and Treatment of Bone and Joint Diseases, Shanghai Institute of Traumatology and Orthopaedics, Ruijin Hospital, Shanghai Jiao Tong University School of Medicine, 197 Ruijin 2nd Road, Shanghai 200025, P. R. China.
E-mail: wgcui80@hotmail.com (W. Cui)

Dr. G. Chen, and Prof. Z. Xiao
Department of Pharmacology and Chemical Biology, & Institute of Molecular Medicine, School of Medicine, Shanghai Jiao Tong University, Shanghai 200025, P. R. China.

Dr. L. Wang
Department of Geriatrics, Ruijin Hospital, Shanghai Jiao Tong University School of Medicine, 197 Ruijin 2nd Road, Shanghai 200025, P. R. China.

Keywords: protein hydrogel, microthrombosis, inflammatory bowel disease

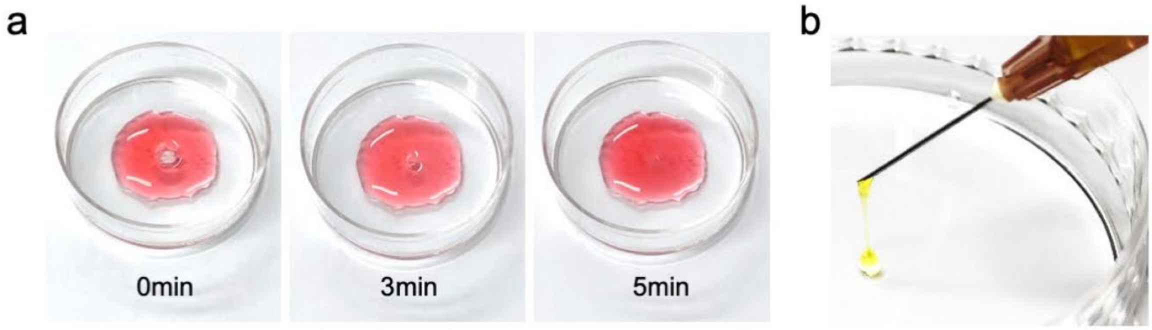

Figure S1. Characteristics of HEP-Ag-BSA. (a) Self-healing property demonstrated by infusion of hydrogel in 5 minutes. (b) Injectability of HEP-Ag-BSA.

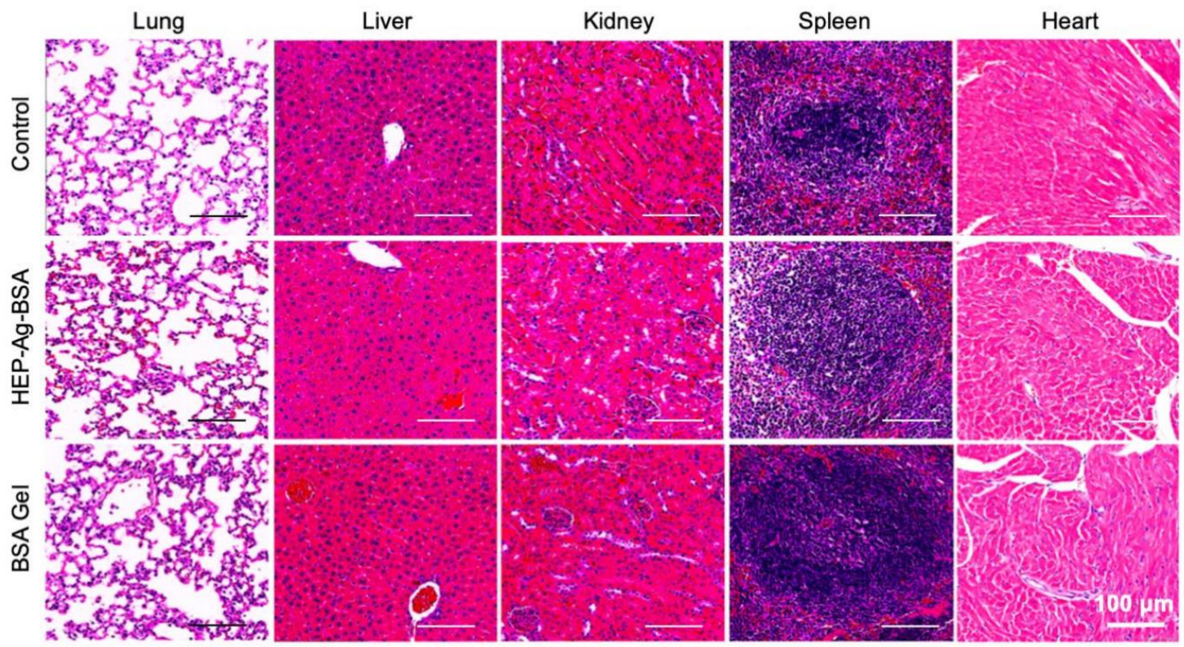

Figure S2. Biocompatibility of hydrogels. Histopathological analyses of sections of vital organs of mice after 8 days of administration of HEP-Ag-BSA and BSA Gel. (scale bar was 100 μm)

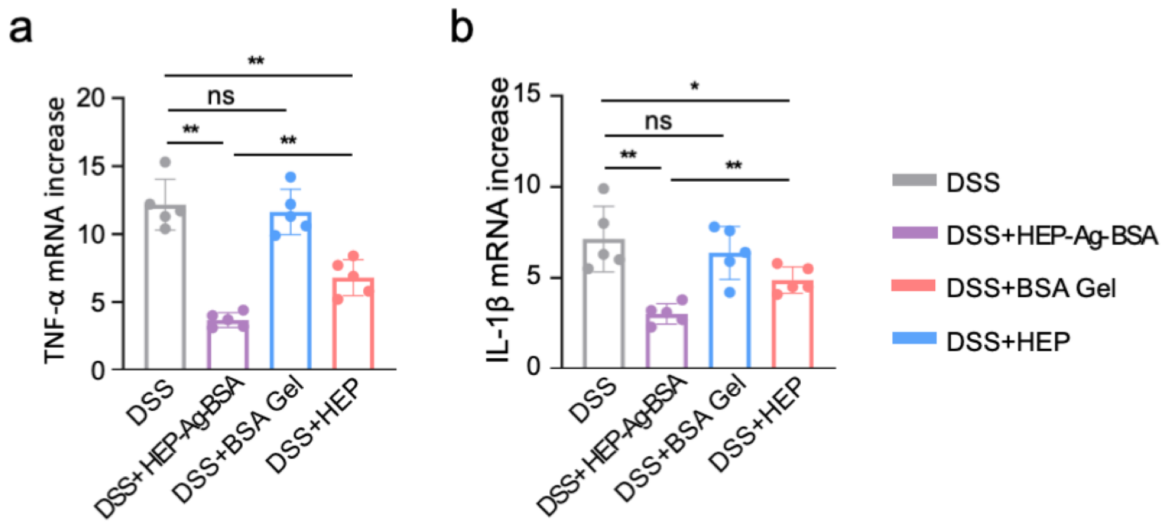

Figure S3. Semi-quantitative analyses of serum mRNA levels of pro-inflammatory factors. (a) mRNA levels of TNF-α. (b) mRNA levels of IL-1β. (n=5; Data were presented as mean ± SD. Significance between every two groups was determined via Mann-Whitney U-test.)

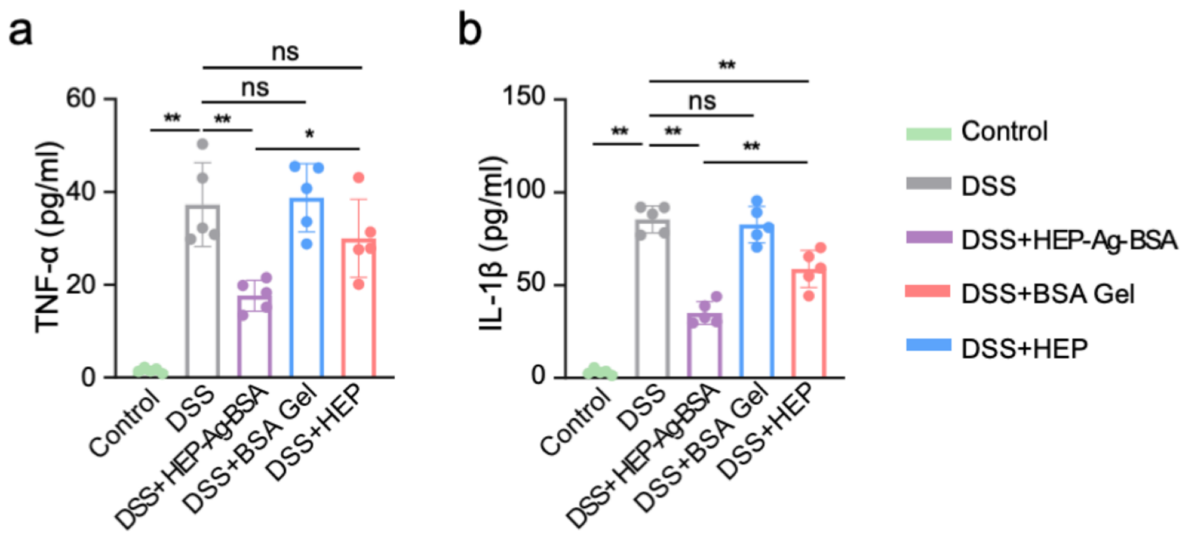

Figure S4. Serum levels of pro-inflammatory factors according to ELISA analysis. (a) Serum levels of TNF-α. b) Serum levels of IL-1β. (n=5; Data were presented as mean ± SD. Significance between every two groups was determined via Mann-Whitney U-test.)
